# P-1919. Incidence of Invasive Group A *Streptococcus* Infection in relation to the COVID-19 Pandemic (GASCO) – a pilot study

**DOI:** 10.1093/ofid/ofae631.2079

**Published:** 2025-01-29

**Authors:** Hannah Bray, Jacob C Stremers, Adam Caulfield, Robert D Fulmer, Zachary Ciochetto, Gordana Simeunovic

**Affiliations:** Corewell Health/Michigan State University, Muskegon, Michigan; Corwell Health West, Byron Center, MI, Michigan; Corewell Health, Grand Rapids, Michigan; Corewell Health West, Grand Rapids, Michigan; Corewell Health, Grand Rapids, Michigan; Corewell Health/ Michigan State University, grand rapids, Michigan

## Abstract

**Background:**

Group A Streptococcus (GAS) is a known cause of invasive infections in humans leading to significant morbidity and mortality. During the COVID-19 pandemic the incidence of respiratory infections had decreased, likely due to pandemic interventions. Previous studies found a correlation between the rates of viral respiratory infections and a predisposition to invasive GAS infections (iGAS). We hypothesize that the incidence of iGAS among adults may have been affected by the COVID-19 pandemic, but evidence is lacking. GASCO compares the rate of iGAS infections before, during and after the COVID-19 pandemic, and evaluates factors that may affect the incidence and outcomes. In this pilot study, we describe the incidence of total GAS infection (tGAS) and iGAS in relation to the COVID-19 pandemic.

Study Flow

**Figure 1.**
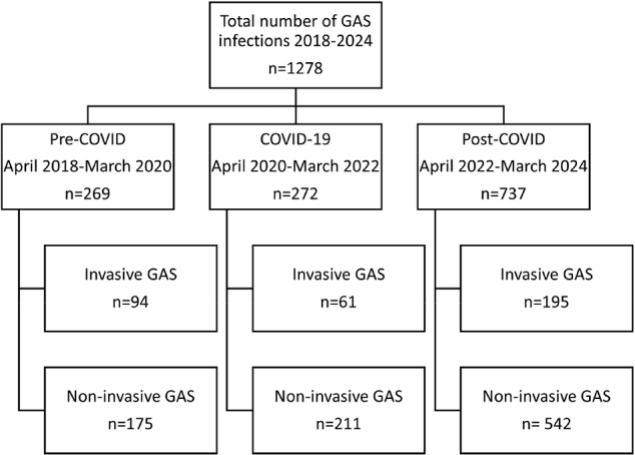
*GAS= Group A Streptococcus infection. **Invasive GAS = Group A Streptococcus isolated from blood, sputum, body fluid, tissue, urine, or cervical culture. ***Non-invasive GAS = Group A Streptococcus isolated from throat or wound culture.

**Methods:**

We retrospectively identified all patients hospitalized at Corewell Health in West Michigan from April 1st, 2018, through March 31st, 2024, with positive GAS culture obtained in the emergency department or hospital. Patients were classified based on the type of infection (invasive versus noninvasive) and time period in relation to COVID-19 (pre-COVID, COVID, and post-COVID) (figure 1). iGAS was defined as being isolated from blood, sputum, body fluid, tissue, urine or cervical culture. Cultures from throat or wound samples were classified as non-invasive.

Number of Total and Invasive Group A Streptococcus Infections during Pre-COVID, COVID and Post-COVID

**Figure 2.**
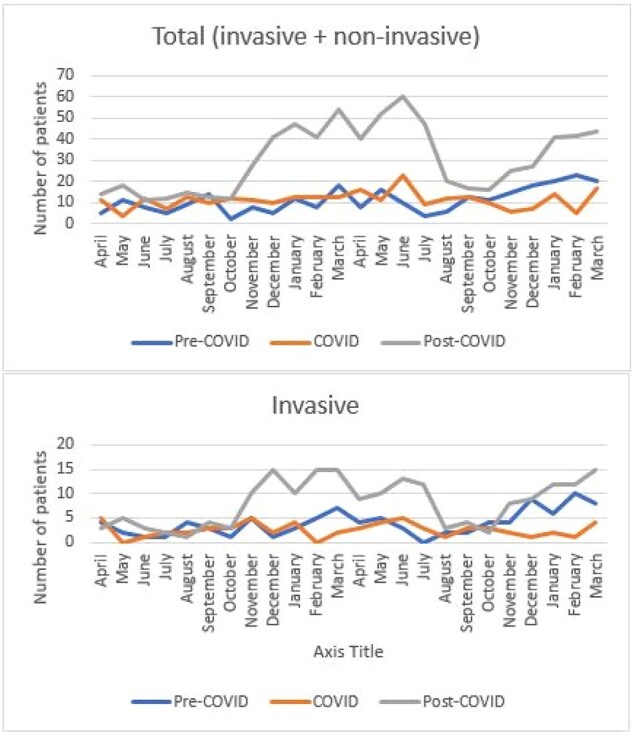
The number of total GAS infections was 2.7 times higher post-COVID than during and pre-COVID. Number of invasive GAS infections post-COVID was 2 times higher than pre-COVID and 3.2 times higher than during the COVID pandemic.

**Results:**

Of 1278 patients who had positive GAS culture between 2018 and 2024, 350 (27%) had iGAS. Absolute number of tGAS and iGAS increased post-COVID compared with COVID and pre-COVID (figure 2), although percentage of tGAS representing iGAS was higher pre-COVID (94/269, 35%) than during COVID (61/272, 22%) or post-COVID (195/737, 26%), p=0.003 (Figure 3). Types of iGAS are shown in figure 4.

**Figure 3.**
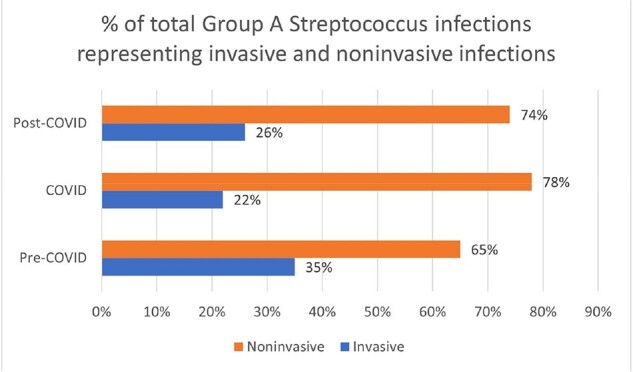
Percentage of total Group A Streptococcus (GAS) infections representing invasive and non-invasive infections during pre-COVID, COVID, and post-COVID periods. Although the absolute number of total and invasive GAS infections was highest post-COVID, the proportion of total GAS infections classified as invasive was highest pre-COVID.

**Conclusion:**

Our findings suggest that the incidence of tGAS and iGAS increased significantly after COVID-19. Some pediatric studies documented an increase in total GAS infections in Europe, possibly due to the pandemic interventions that led to a gap in immunity and increased susceptibility to infection, and expansion of strains with increased fitness. The full study will evaluate iGAS outcomes and address the factors impacting incidence and outcomes, aiming to provide better insight into the epidemiology of iGAS infections.

**Figure 4.**
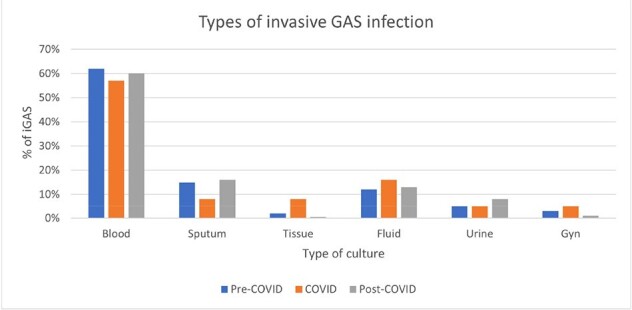
Types of invasive Group A Streptococcus infection during pre-COVID, COVID, and post-COVID periods. Blood cultures were the most frequently positive throughout the whole study period. There was no significant difference in the types of positive cultures between periods.

**Disclosures:**

All Authors: No reported disclosures

